# Supplementary material for: Molecular Traits and Functional Exploration of BES1 Gene Family in Plants
Source: Int J Mol Sci. 2022 Apr 11;23(8):4242. doi: 10.3390/ijms23084242 (PMC9027564; doi:10.3390/ijms23084242)
Supplement: Supplementary file 1 [file ijms-23-04242-s001.zip › ijms-1644054suppl for proof.pdf]

Table S1 Physical and chemical property of *BES1* genes in plants

Lineages	Species	Locus Name	A. A.	*E* value	Chromosomal locations	Reference
Bryophytes	*Physcomitrella patens*	XP_024362740.1	356	3.7E-55	XM_024506972.1: 1825...2895	This study
		XP_024357987.1	358	3.7E-55	XM_024502219.1: 1557...2633	This study
		XP_024391346.1	349	3.7E-55	XM_024535578.1: 1626...2675	This study
		XP_024373852.1	357	2.8E-38	XM_024518084.1: 1393...2466	This study
		XP_024375445.1	501	2.8E-38	XM_024519677.1: 1518...3023	This study
		XP_024377600.1	509	2.8E-38	XM_024521832.1: 1997...3526	This study
	Marchantia polymorpha	PTQ45822.1	352	2.0E-34	KZ772685.1: 507763...508491	This study
		PTQ44678.1	404	5.9E-50	KZ772691.1: 863385...864383	This study
		PTQ35275.1	610	7.1E-44	KZ772744.1: 283976...284415	This study
		PTQ35274.1	599	2.0E-44	KZ772744.1: 283976...284415	This study
Lycophytes	*Selaginella moellendorffii*	Sm437216	530	1.2E-44	scaffold_0:304558-306242 reverse	This study
		Sm407084	358	1.2E-56	scaffold_6:2163227-2164361 reverse	This study
		Sm429765	331	5.1E-58	scaffold_119:338603-339695	This study
		Sm414095	332	9.8E-06	scaffold_22:1553181-1554327 reverse	This study
Gymnosperms	*Picea abies*	MA_93796g0010	199	2.6E-06	MA_93796:19611-21078	This study
		MA_11814g0010	279	7.9E-53	MA_11814:36845-37767	This study
		MA_482543g00010	333	2.4E-58	MA_482543:909-1910	This study
		MA_195595g0010	276	2.5E-58	MA_195595:7971-8893	This study
		MA_245615g0010	311	1.2E-53	MA_245615:6633-7795	This study
	Picea sitchensis	ADE77805.1	302	2.5E-49	BT124563.1: 143...1051	This study
		ADE77262.1	305	5.7E-46	BT123979.1: 46...963	This study
		ABK21940.1	367	1.8E-65	EF082584.1: 144...1247	This study
		ABR18463.1	407	1.0E-64	EF678735.1: 155...1378	This study

Bacal angiosperm	*Nymphaea colorata*	XP_031504621.1	333	4.1E-67	XM_031648761.1: 1804...2805	This study
		XP_031504620.1	333	4.1E-67	XM_031648760.1: 1887...2888	This study
		XP_031495999.1	356	5.9E-66	XM_031640139.1: 299...1369	This study
		XP_031495998.1	357	2.9E-64	XM_031640138.1: 298...1371	This study
		XP_031480198.1	389	2.9E-64	XM_031624338.1: 328...1497	This study
		XP_031505422.1	374	1.2E-34	XM_031649562.1: 919...2043	This study
		XP_031483344.1	721	4.1E-39	XM_031627484.1: 908...3073	This study
		XP_031483343.1	721	4.1E-39	XM_031627483.1: 1201...3366	This study
		XP_031502181.1	661	9.5E-45	XM_031646321.1: 138...2123	This study
	Amborella trichopoda	ERM94797.1	700	2.0E-48	KI397507.1: 8556991...8557091	This study
		ERN17571.1	143	2.0E-50	KI392312.1: 1925088...1925342	This study
		XP_006827627.2	687	2.0E-40	XM_006827564.3: 971...3034	This study
		XP_006829668.1	204	3.8E-44	XM_006829605.3: 2362...2976	This study
		XP_006833072.1	293	5.8E-67	XM_006833009.3: 1380...2261	This study
		XP_006856105.2	327	8.9E-60	XM_006856043.3: 541...1524	This study
		XP_011624011.1	676	1.9E-48	XM_011625709.2: 424...2454	This study
		XP_011624018.1	668	1.8E-48	XM_011625716.2: 426...2432	This study
		XP_020523933.1	274	2.4E-40	XM_020668274.1: 552...1376	This study
Monocots	*Zea mays*	NP_001132634.1	345	2.8E-38	NM_001139162.1: 247...1284	This study
		NP_001136786.1	355	2.8E-38	NM_001143314.1: 337...1404	This study
		NP_001151195.2	317	1.1E-64	NM_001157723.2: 169...1122	This study
		NP_001168185.1	378	5.0E-52	NM_001174714.1: 77...1213	This study
		NP_001336096.1	324	5.0E-52	NM_001349167.1: 133...1107	This study
		XP_008645603.3	363	5.0E-52	XM_008647381.3: 4...1095	This study
		NP_001337631.1	679	5.0E-52	NM_001350702.1: 214…2253	This study

		NP_001357917.1	171	5.0E-52	NM_001370988.1: 160...675	This study
	Sorghum bicolo	Sobic.002G353200.1	336	1.7E-42	Chr02:71635214-71637226 reverse	This study
		Sobic.001G511400.1	412	1.5E-53	Chr01:77886570-77891227 reverse	This study
		Sobic.003G026300.1	348	1.2E-24	Chr03:2228809-2231971 forward	This study
		Sobic.004G1025001	399	1.9E-21	Chr04:9584881-9586747 reverse	This study
		Sobic.004G102700.1	375	2.2E-55	Chr04:9632195-9633870 reverse	This study
		Sobic.002G136200.2	596	3.4E-50	Chr02:20563849-20574672 forward	This study
		Sobic.004G027800.2	716	3.9E-39	Chr04:2235985-2242049 forward	This study
		Sobic.010G163900.1	356	5E-73	Chr10:48237055-48240678 forward	This study
	Oryza sativa	Os07g39220	298	2.2E-42	chr07:23483973-23485307	This study
		Os06g35900	355	2.2E-52	chr06:20941120-20944077	This study
		Os02g03690	801	7.1E-21	chr02:1539410-1547341	This study
		Os01g10610	365	5.8E-12	chr01:5669291-5671467	This study
		Os02g13900	376	2.6E-11	chr02:7548547-7551644	This study
		Os01g08180	161	3.9E-52	chr01:3958628-3963625	This study
	Brachypodium distachyum	XP_003576871.1	690	1.2E-38	XM_003576823.4: 276...2348	This study
		XP_014755446.1	659	4.8E-52	XM_014899960.2: 163...2142	This study
		XP_010233755.2	658	4.7E-86	XM_010235453.3: 163...2139	This study
		XP_014753965.1	138	4.8E-25	XM_014898479.2: 1...417	This study
		XP_003563774.1	355	3.7E-74	XM_003563726.4: 193...1260	This study
		XP_003571150.2	378	1.2E-57	XM_003571102.4: 84...1220	This study
		XP_003565213.2	355	1.7E-67	XM_003565165.3: 190...1257	This study
		XP_003562829.1	346	1.0E-66	XM_003562781.4: 127...1167	This study
Eudicots	*Arabidopsis thaliana*	AT1G19350.3	357	2.4E-54	Chr1: 6688535-6690411	This study
		AT1g78700.1	325	4.5E-55	Chr1:29599349-29601682	This study

	AT1G75080.1	336	3.2E-54	Chr1:28185504-28187410	This study
	AT2G45880.1	691	4.1E-55	Chr2:18878518-18882995	This study
	AT3g50750.1	276	5.0E-58	Chr3:18861749-18863397	This study
	AT4G36780.1	265	6.9E-54	Chr4:17332269-17334355	This study
	AT4G18890.1	284	1.3E-65	Chr4:10352386-10355555	This study
	AT5G45300.1	689	6.3E-48	Chr5: 18353610-18356886	This study
Gossypium arboreum	Ga01G0527.1	325	1.80E-70	Chr01:6346471-6351159	This study
	Ga03G1163.1	313	1.90E-65	Chr03:52996397-52997611	This study
	Ga05G2078.1	329	3.70E-66	Chr05:18998585-19000722	This study
	Ga05G2760.1	314	2.10E-64	Chr05:28055825-28057353	This study
	Ga06G2442.1	298	1.10E-70	Chr06:130288229-130293237	This study
	Ga08G1570.1	703	3.80E-42	Chr08:104646255-104652458	This study
	Ga10G2726.1	319	5.20E-64	Chr10:126361581-126365738	This study
	Ga11G0547.1	317	1.50E-61	Chr11:8086113-8089296	This study
	Ga04G0571.1	178	6.15E-24	Chr04: 10250819-10251435	This study
	Ga11G3133.1	708	1.10E-41	Chr11:116053478-116061911	This study
	Ga12G0797.1	660	4.90E-55	Chr12:7486165-7492139	This study
Gossypium hirsutum	Gh_A01G0360	325	2.10E-70	A01:5334151-5337014	This study
	Gh_A03G1024	313	1.90E-65	A03:70746333-70747547	This study
	Gh_A04G0814	178	1.10E-37	A04:53859398-53860014	This study
	Gh_A05G1683	313	3.60E-66	A05:17518565-17519929	This study
	Gh_A05G2217	314	9.20E-65	A05:25604609-25606137	This study
	Gh_A06G1990	322	9.00E-71	scaffold1329_A06:23097-26810	This study
	Gh_A10G0312	319	5.20E-64	A10:2874590-2876941	This study
	Gh_A11G2598	317	1.90E-62	A11:86626967-86630153	This study

	Gh_D01G0390	325	6.80E-70	D01:4645236-4648082	This study
	Gh_D02G0939	313	7.00E-66	D02:21203790-21205026	This study
	Gh_D04G1312	176	2.80E-38	D04:42765947-42766557	This study
	Gh_D05G1874	313	3.20E-66	D05:17106711-17108075	This study
	Gh_D05G2477	314	2.90E-64	D05:24906886-24908412	This study
	Gh_D06G2134	322	1.60E-69	D06:63169839-63173384	This study
	Gh_D08G1459	703	4.10E-42	D08:47703731-47709874	This study
	Gh_D10G0318	319	5.20E-64	D10:2754452-2756809	This study
	Gh_D11G2965	317	1.50E-60	D11:60501052-60504323	This study
	Gh_D11G0932	737	1.10E-41	D11:8079122-8088177	This study
	Gh_A08G1175	703	4.00E-43	A08:81628093-81634232	This study
	Gh_D12G1996	657	1.90E-54	D12:52951020-52956781	This study
	Gh_A12G1824	659	4.90E-86	A12:95393587-95400098	This study
	Gh_A11G0794	693	1.50E-41	A11:8277495-8284009	This study
Gossypium raimondii	Gorai.009G274000.1	314	4.7E-25	Chr09:22931216-22933366 reverse	This study
	Gorai.009G204600.1	313	7.7E-24	Chr09:15827455-15829456 reverse	This study
	Gorai.005G107000.1	313	3.7E-55	Chr05:19206384-19208322 reverse	This study
	Gorai.007G327000.1	317	7.7E-45	Chr07:54634447-54638641 reverse	This study
	Gorai.010G240700.1	322	7.1E-54	Chr10:61004462-61008499 reverse	This study
	Gorai.002G052400.1	325	5.2E-24	Chr02:4601377-4605470 forward	This study
	Gorai.011G036300.1	319	5.9E-25	Chr11:2725969-2729103 reverse	This study
	Gorai.012G118900.1	176	1.2E-53	Chr12:27286831-27287441 reverse	This study
	Gorai.007G099100.1	699	9.9E-50	Chr07:7317323-7324017 reverse	This study
	Gorai.004G158600.1	714	7.5E-22	Chr04:44777016-44784041 reverse	This study
	Gorai.008G218300.1	655	3.2E-52	Chr08:50463840-50470692 forward	This study

Aquilegia coerulea	Aqcoe1G215900.1	366	4.2E-42	Chr_01:12959004-12962929 reverse	This study
	Aqcoe4G027100.1	328	5.2E-52	Chr_04:2339145-2342855 reverse	This study
	Aqcoe6G111000.1	313	5.1E-25	Chr_06:5978807-5980444 forward	This study
	Aqcoe7G033500.1	736	3.8E-12	Chr_07:1989998-1996859 forward	This study
Mimulus guttatus	Migut.C00690.1	333	1.6E-13	scaffold_3:14809125-14811043 forward	This study
	Migut.N00514.1	325	2.8E-52	scaffold_14:2530550-2532452 reverse	This study
	Migut.H00192.1	304	1.4E-24	scaffold_8:1026675-1028366 reverse	This study
	Migut.M00715.1	324	1.5E-52	scaffold_13:14806288-14809280 forward	This study
	Migut.M00997.1	337	1.2E-53	scaffold_13:16430712-16433959 reverse	This study
	Migut.J00102.1	158	1.1E-52	scaffold_10:475367-475908 forward	This study
	Migut.N01443.1	704	2.0E-52	scaffold_14:12020884-12026746 reverse	This study
	Migut.A00486.1	681	2.1E-53	scaffold_1:2538656-2542470 reverse	This study

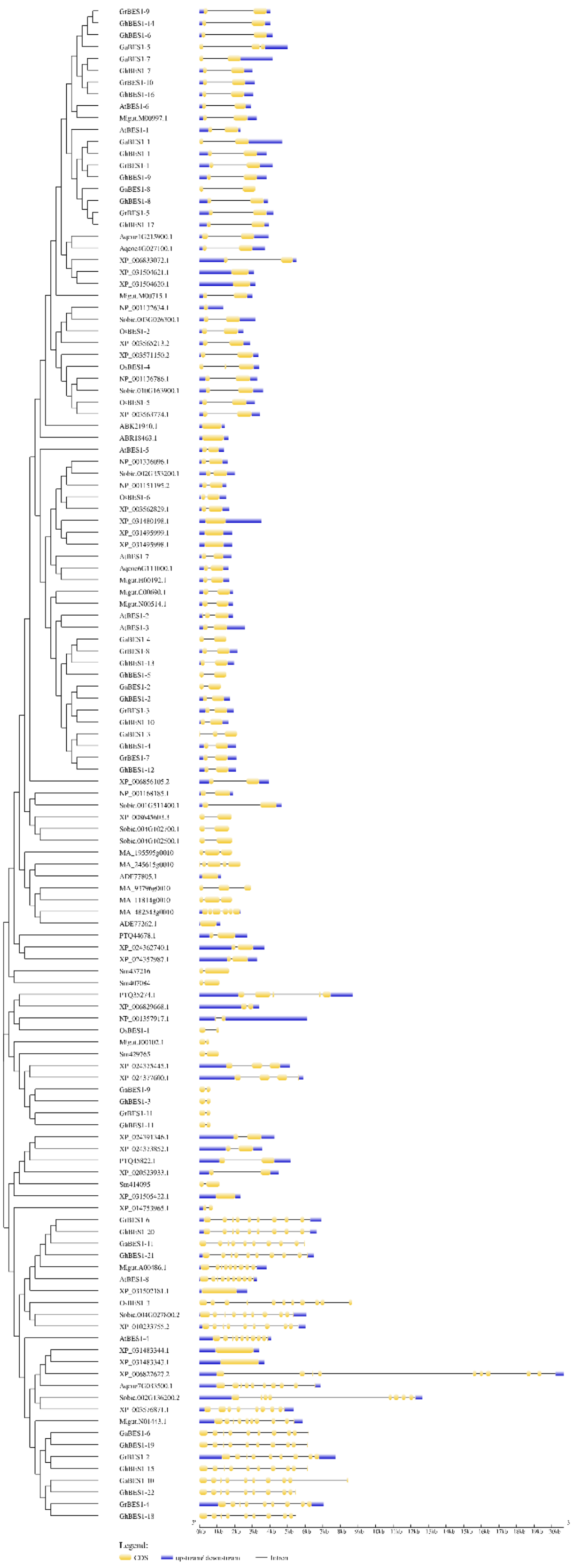

Figure. S1 Map of gene structure of *BES1* gene, ▬ represent CDS, ——represent Intron.

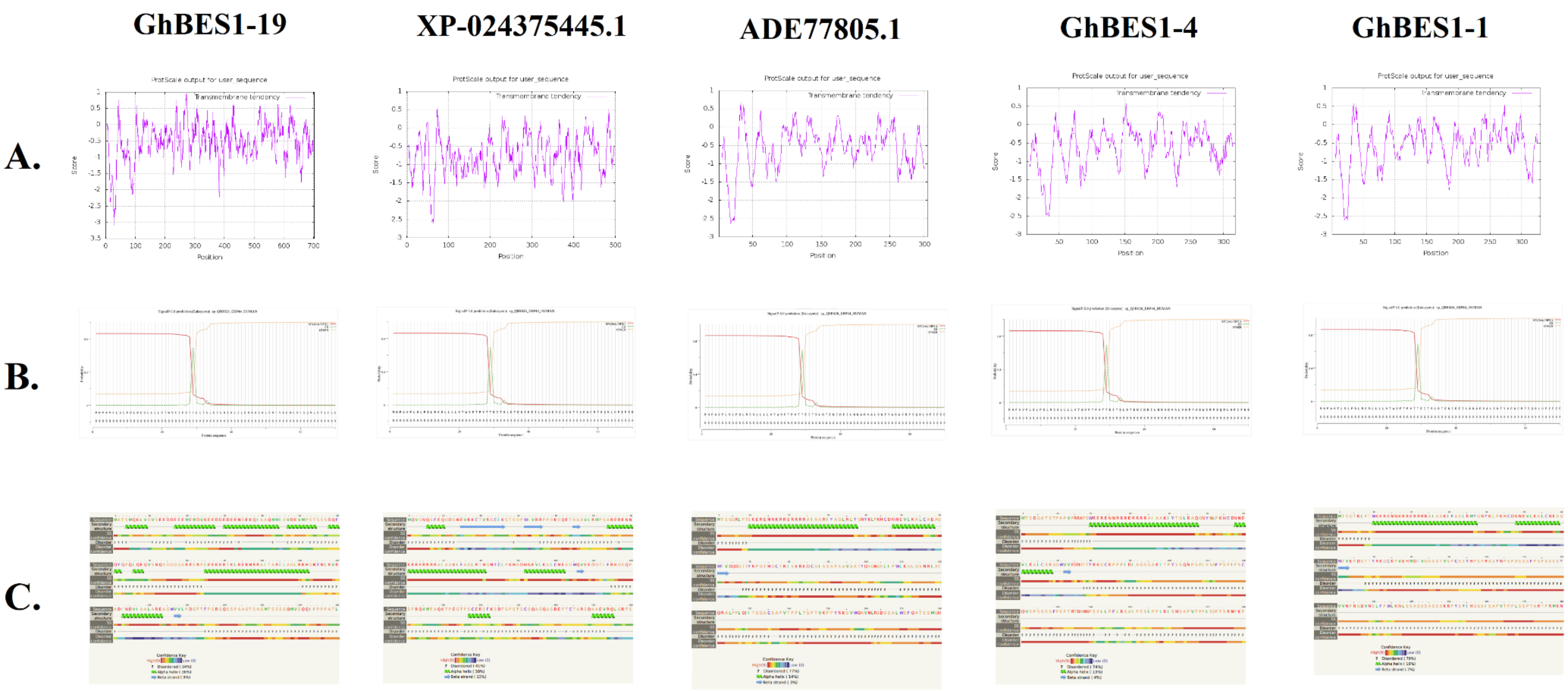

Figure. S2 Characterization of BES1 proteins in five subfamilies A. Hydrophilic and hydrophobic analysis B. Signal peptide detection C. Secondary structure analysis

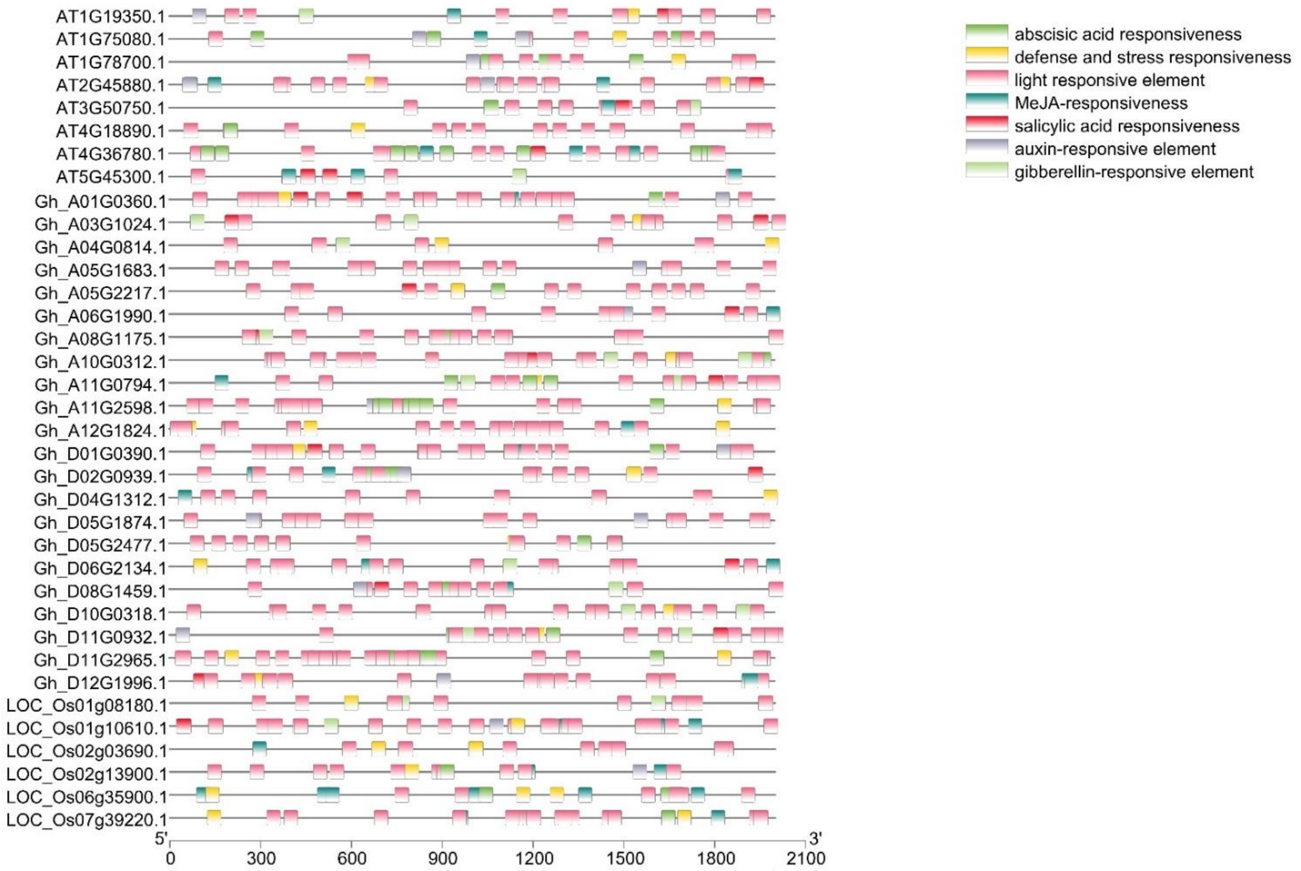

Figure. S3 *Cis*-acting element analysis of *BES1* gene in Arabidopsis, rice and cotton, include abscisic acid, auxin, defense and stress, gibberellin, and MeJA have been recognized, implying their regulation in hormones or stress responses.

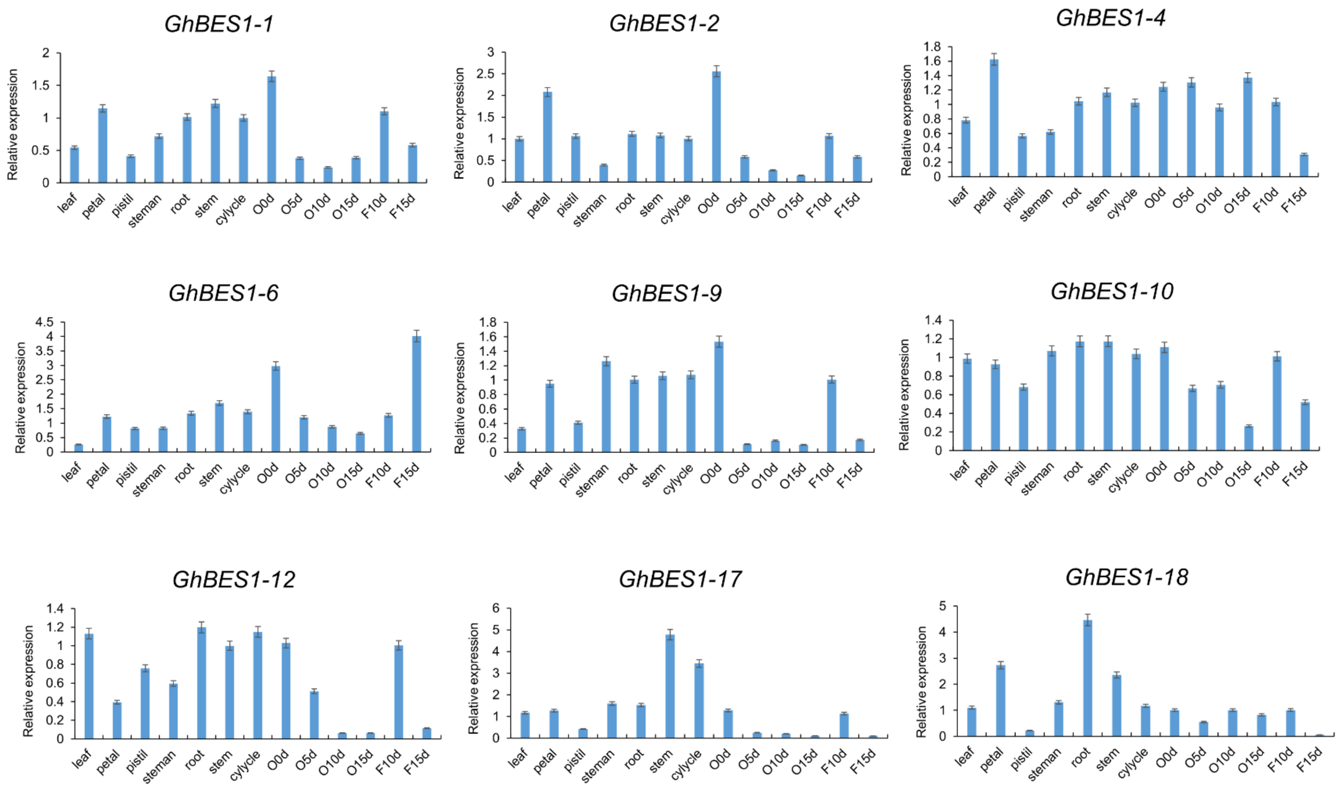

Figure. S4 Expression patterns of 9 *GhBES1s* in tissues, leaf, petal, pistil, root, stem, cylycle, ovule and fiber based on qRT-PCR. Three independent experiments were performed. Error bar represents SD.

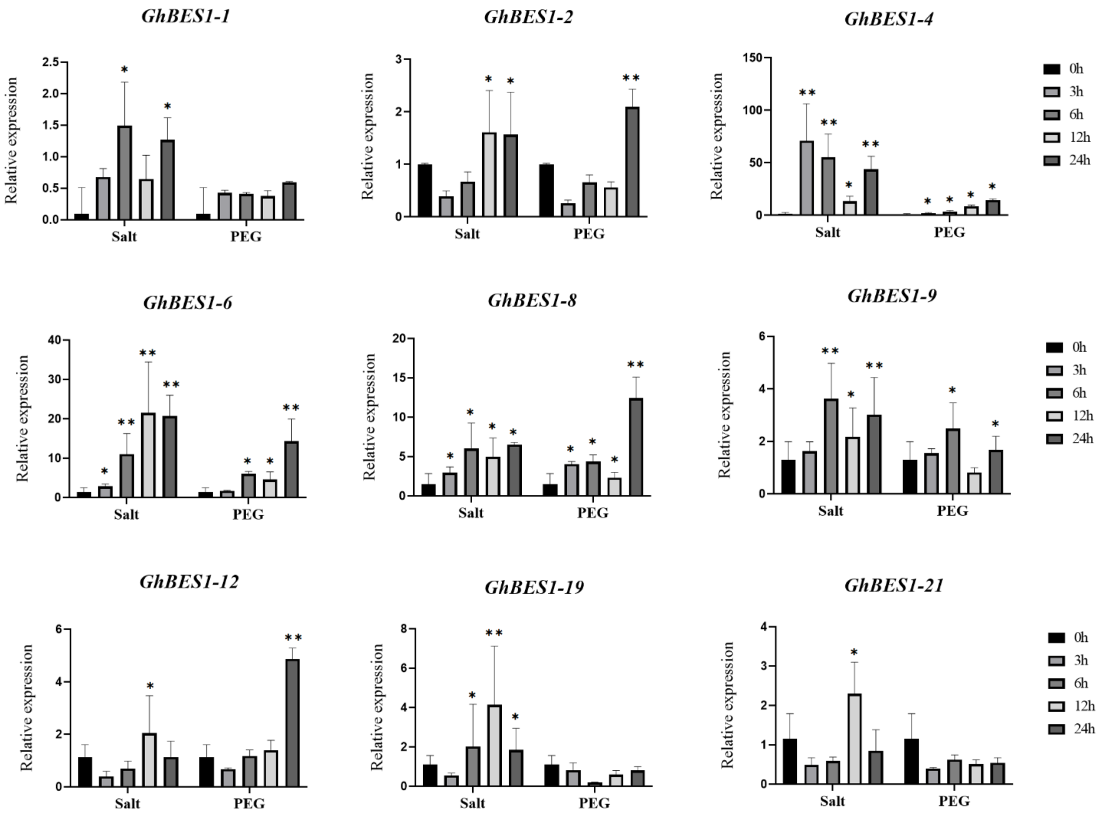

Figure. S5 Expression patterns of 9 *GhBES1s* under salt and PEG stress in 0h, 3h, 6h, 12h and 24h based on qRT-PCR. Three independent experiments were performed. *: $0.01<p<0.05$, **: $p<0.01$. Error bar represents SD.

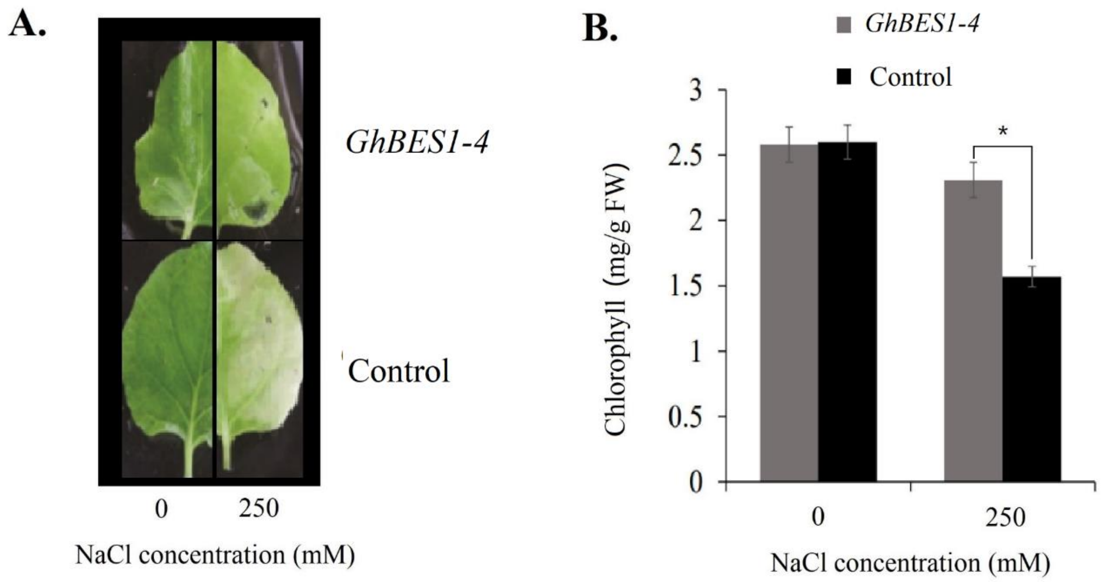

Figure. S6 A. Phenotypic analysis of transgenic tobacco and control under NaCl treatment. B. Measurements of chlorophyll content of detached leaves (mg/g fresh weight). Data were mean ± SE of three replicates with each replicate containing ten plants. The '*' indicates $p<0.05$. Error bar represents SD.
